# Supplementary material for: Harderian Gland Development and Degeneration in the Fgf10-Deficient Heterozygous Mouse
Source: J Dev Biol. 2024 Jun 3;12(2):16. doi: 10.3390/jdb12020016 (PMC11205083; doi:10.3390/jdb12020016)
Supplement: Supplementary file 1 [file jdb-12-00016-s001.zip › jdb-2959290-supplementary.pdf]

Supplementary Information for

Harderian gland development and degeneration in the *Fgf10*-deficient heterozygous mouse

Shiori Ikeda 1, Keita Sato 2, Hirofumi Fujita 2, Hitomi Ono-Minagi 3, Satoru Miyaishi 4, Tsutomu Nohno 1, Hideyo Ohuchi 2*

1 Department of Cytology and Histology, Okayama University Medical School, 2-5-1 Shikata-cho, Kita-ku, Okayama 700-8558, Japan
2 Department of Cytology and Histology, Okayama University Faculty of Medicine, Dentistry and Pharmaceutical Sciences, 2-5-1 Shikata-cho, Kita-ku, Okayama 700-8558, Japan
3 Department of Cytology and Histology, Okayama University Graduate School of Medicine, Dentistry and Pharmaceutical Sciences, 2-5-1 Shikata-cho, Kita-ku, Okayama 700-8558, Japan
4 Department of Legal Medicine, Okayama University Faculty of Medicine, Dentistry and Pharmaceutical Sciences, 2-5-1 Shikata-cho, Kita-ku, Okayama 700-8558, Japan

*Correspondence: ohuchi-hideyo@okayama-u.ac.jp

Supplementary Methods

Quantitative PCR (qPCR)

Embryonic heads at E19 were dissected, divided into right and half halves, and immediately dipped in RNAlater solution (Thermo Fisher Scientific). After 3 days at 4 degrees, RNAlater was removed, and the samples were stored at -80 degrees. When RNA was extracted, NucleoZol solution was added to the frozen tissues, homogenized with a Power Macher II (nippi; Fujifilm Wako, Osaka, Japan), and processed for RNA isolation. cDNA synthesis and qPCR (Fig. S10A, B) were performed as below.

As for E15 samples (Fig. S10C), PFA-fixed embryonic bodies (WT, n = 2; *Fgf10*$^{+/-}$, n = 3; *Fgf10*$^{-/-}$, n = 3), frozen for cryosectioning were thawed and washed in PBS three times. The embryonic bodies were homogenized in 1x Protection Reagent (NEB, Ipswich, MA) with a Power Macher II. Total RNA was extracted and purified from 25 mg of homogenized tissues using Monarch Total RNA Miniprep Kit (NEB). Since the tissues were fixed in PFA, proteinase K treatment was performed sufficiently to liberate nuclei acids according to the manufacturer's protocol. After confirmation of RNA integrity, cDNA was synthesized from 1 ug of total RNA in 20 uL volume using FastGene™ Scriptase II (NE-LS65; Nippon Genetics, Tokyo, Japan). After 1:5 dilution, 1 uL of the cDNA solution was used for qPCR in 15 uL volume using THUNDERBIRD Next SYBR™ qPCR Mix (QPX-201; TOYOBO, Osaka, Japan) on LightCycler Nano (Roche, Basel, Switzerland).

As for 19-week Harderian glands (HGs) (Fig. S10D), the tissues were dissected out from the eyeball and immediately dipped in RNA later solution (Thermo Fisher Scientific). The procedures for sample storage, RNA isolation with Nucleozol, cDNA synthesis and qPCR were performed as the same as the above.

qPCR primer sequences and amplicon size are listed in Table S7. Statistical analysis was performed with Igor Pro 9 software (https://www.wavemetrics.com/products/igorpro) version 9.0.2.4 (WaveMetrics). The source data for qPCR (Fig. S10) is shown in Table S8.

Supplementary Figures

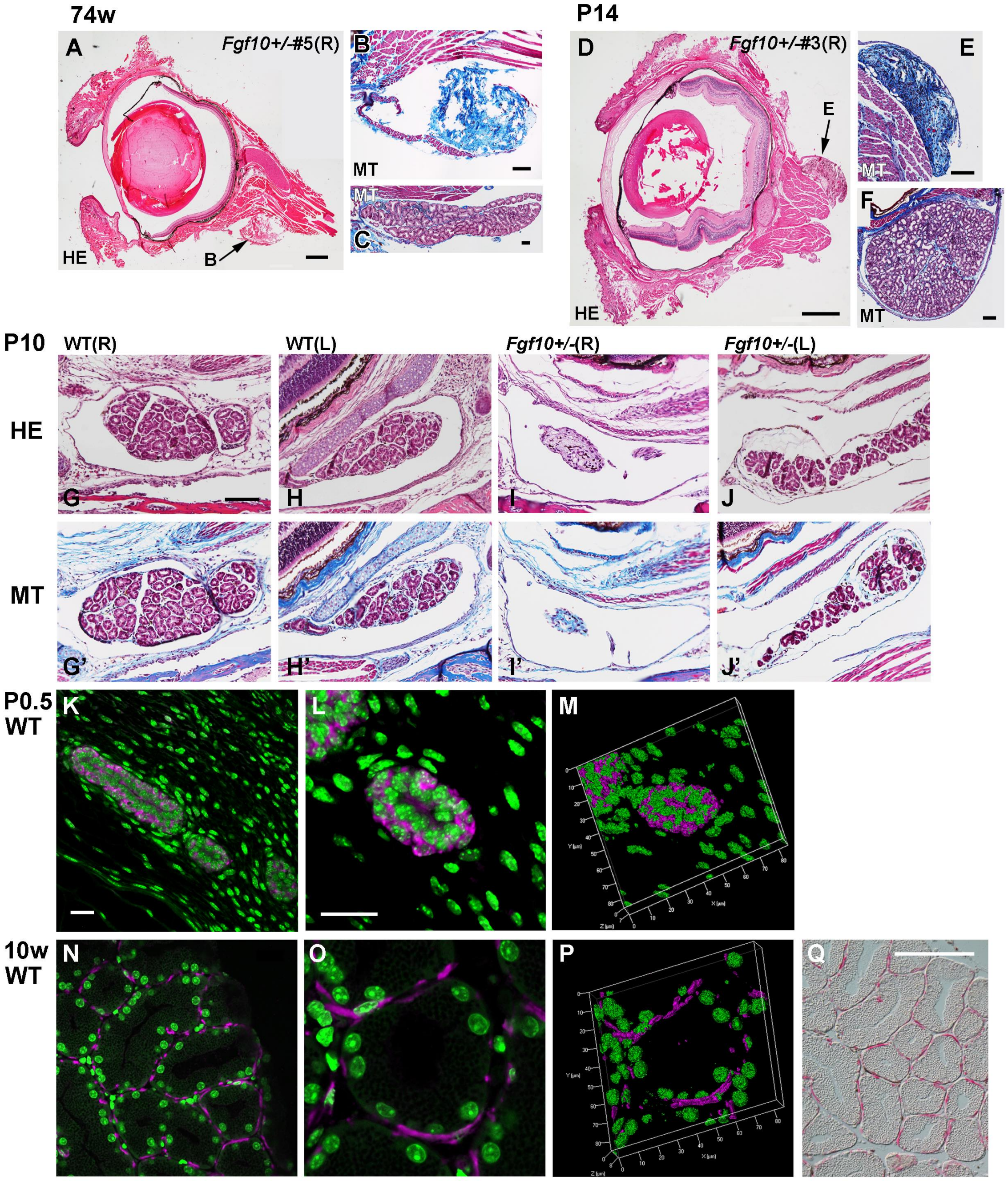

Figure S1. Harderian gland phenotype of the *Fgf10* heterozygous knockout mouse (related to Figure 1): The unilateral gland is often degenerated within 2 weeks from birth. (**A-F**) Histology of *Fgf10*$^{+/-}$ eyes with HGs (arrows) of 74-week-old (**A-C**) and P14 (**D-F**) mice. Hematoxylin-eosin (HE) (**A, D**) or Masson trichrome (MT) (**B, C, F, F**) stain was performed. Right (**A, B, D, E**) and left (**C, F**) sides are shown. Marked fibrosis is observed in (**B, E**). (**G-J, G′-J′**) Histology of the developing HGs of WT and *Fgf10*$^{+/-}$ mice at P10. Sections of right (**G, G′, I, I′**) and left (**H, H′, J, J′**) HGs are shown. HE stain (**G-J**) and corresponding MT stain (**G′-J′**) were performed. (**K-Q**) Immunohistochemistry of pancytokeratin. Panels (**K-P**) show localization of pancytokeratin (magenta) and nuclei (green) in WT P0.5 (**K-M**) or 10-week (**N-P**) HGs as revealed by confocal microscopy (**K, L, N, O:** Z-stack images; **M, P:** 3D images). Panel (**Q**) shows a differential interference contrast (DIC) microscopic image of the HG tissue at 10 weeks. Scale bars: 0.4 mm (**A, D**), 100 µm (**B, C, E, F, G-J, G′-J′, Q**), 20 µm (**K, N, L, O**).

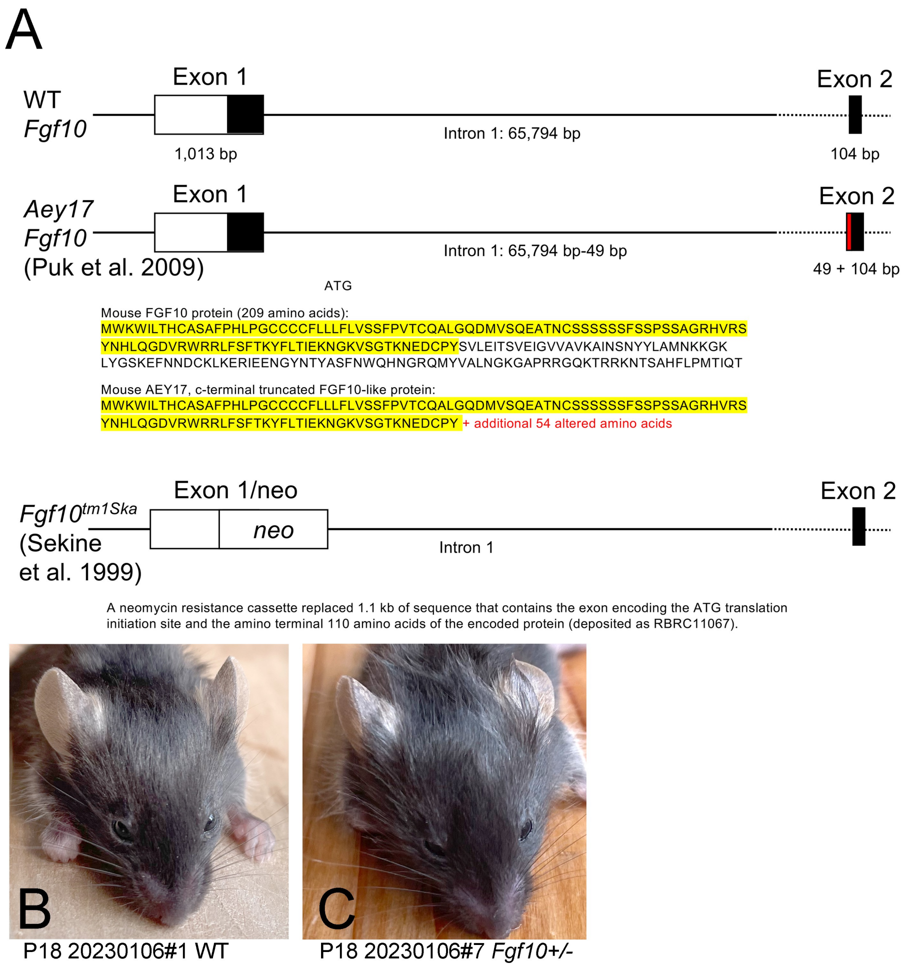

Figure S2. Structure of wild-type and mutant *Fgf10* exons (A) and photos of the face of wild-type (B) and *Fgf10*$^{+/-}$ (C) mice at P18 in this study. (**A**) The mouse *Fgf10* gene has three exons, and the schema depicts the first two exons. The length of exon 1 and intron 1 is after the Ensembl database (ENSMUST00000022246.9).

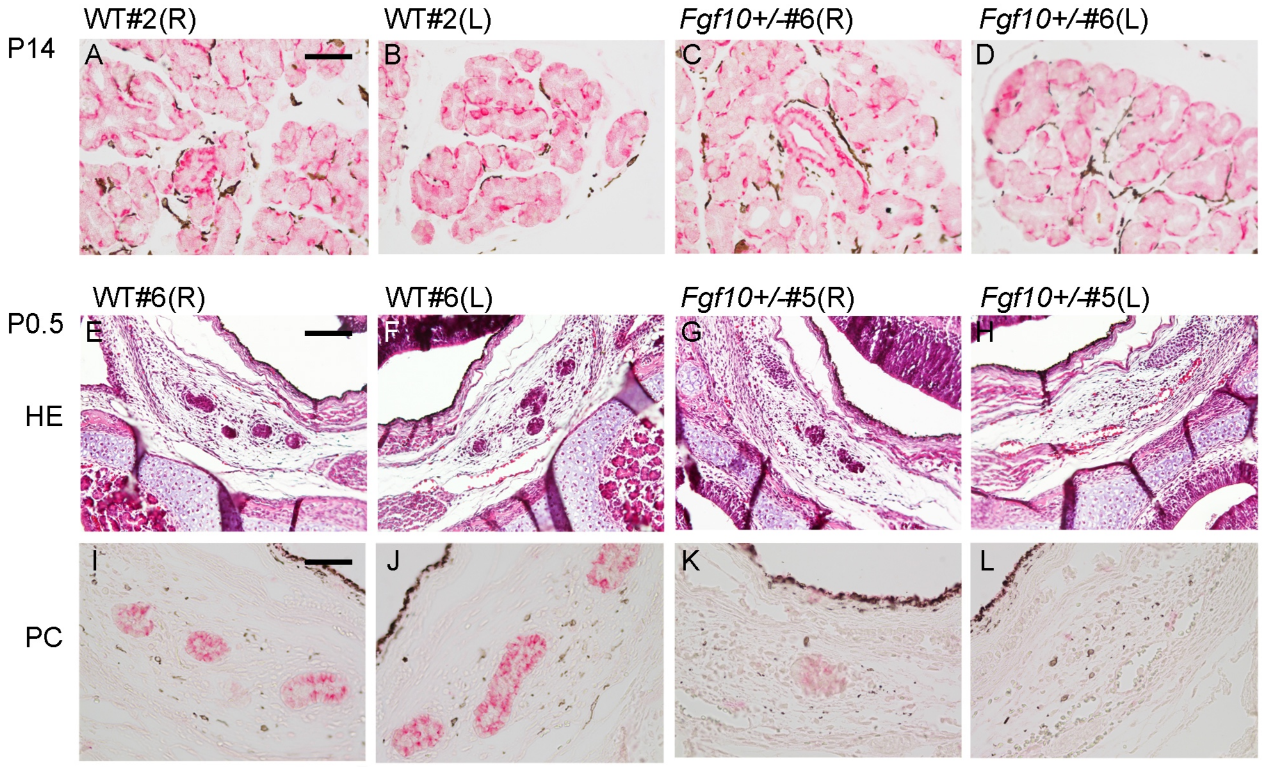

Figure S3. Immunohistochemistry of pancytokeratin, related to Figures 1 and 2. (**A-D**) Sections of HGs on both sides from a WT (**A, B**) and an *Fgf10*$^{+/-}$ (**C, D**) mice at P14 were used. Localization of pancytokeratin in HGs is seen in red. Interstitial melanocytes are observed in dark brown. (**E-H**) Histology (HE stain) of a WT and an *Fgf10*$^{+/-}$ developing HGs of P0.5 mice as indicated. (**I-L**) Immunohistochemistry of pancytokeratin (shown in red). Sections of HGs on both sides from the WT (**E, F, I, J**) and the *Fgf10*$^{+/-}$ (**G, H, K, L**) mice were used. Scale bars: 50 μm (**A-D, I-L**), 100 μm (**E-H**).

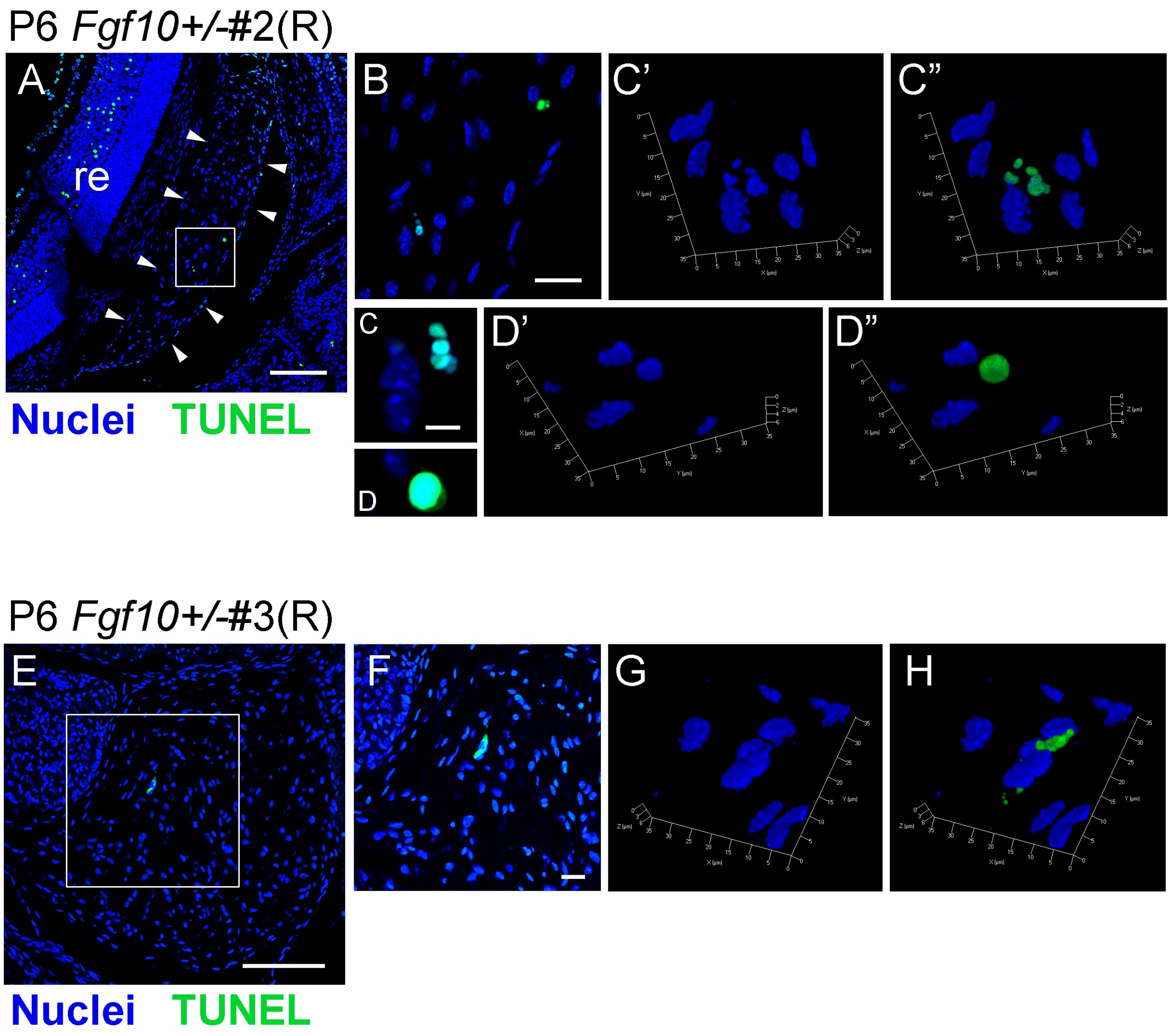

Figure S4. Additional and original data for Fig. 1Z-AA′. Since confocal microscopic images were taken with an inverted light microscope, the photos were mirror-imaged as shown here. Fig. 1AA is shown as the original one, panel H. Boxed area in (**A**) is enlarged in (**B**), where two TUNEL-positive portions are observed. The left is enlarged in (**C-C″**), and the right is enlarged in (**D-D′**). TUNEL-positive signals (green) are merged with fragmented (**C′, C″**) or pyknotic (**D′, D"**) nuclei (blue). Boxed area in (**E**) is enlarged in (**F**). TUNEL positive signal (green in **H**) is partly merged with fragmented nuclei (blue in **G**). Scale bars: 50 μm (**A**), 20 μm (**B, F**), 5 μm (**C, D**), 100 μm (**E**).

P6 *Fgf10+/-*#2R Image C series

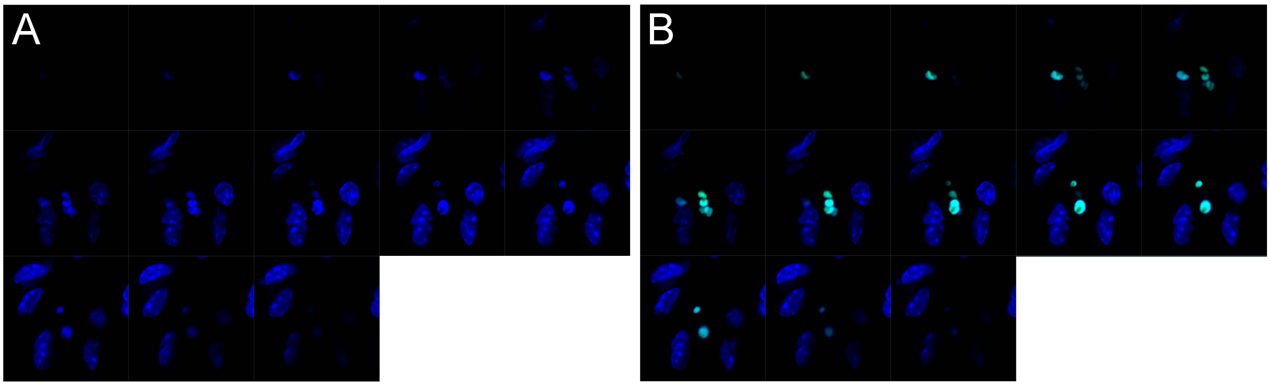

P6 *Fgf10+/-*#2R Image D series

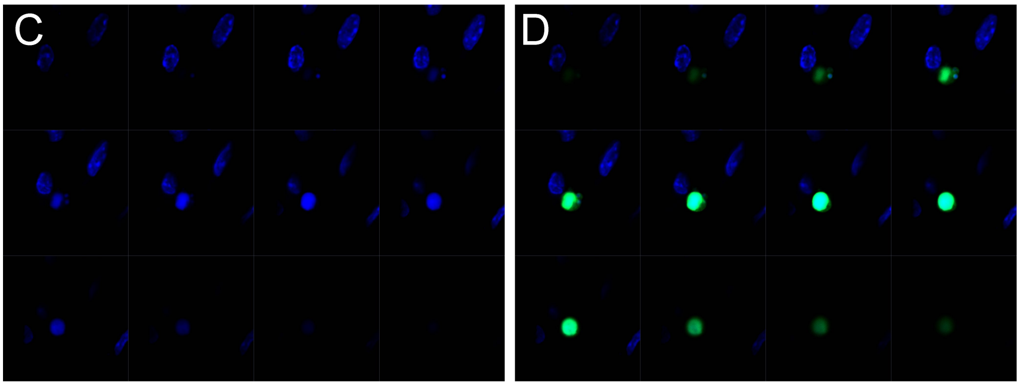

P6 *Fgf10+/-*#3R Images G and H

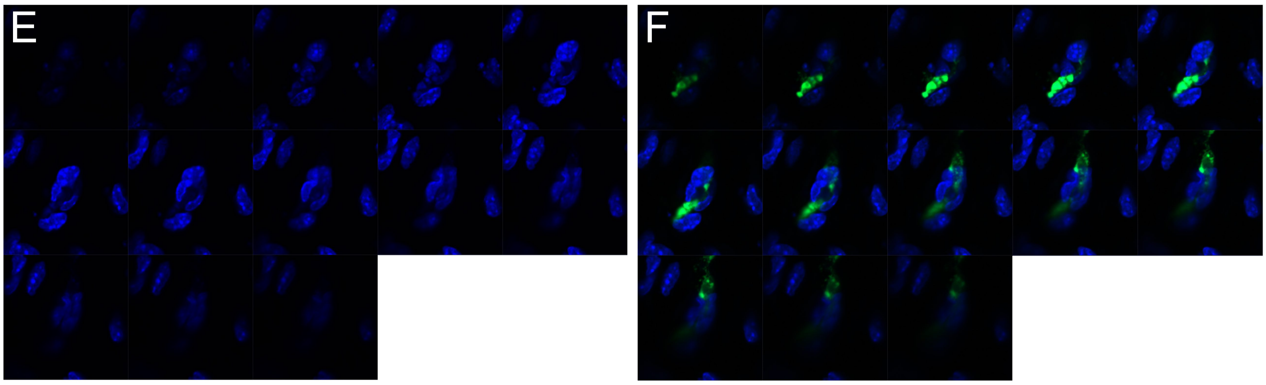

Figure S5. Original source data for Fig. 1Z′, Z″, AA′, and Fig. S4C-C′, D-D″, G, H.

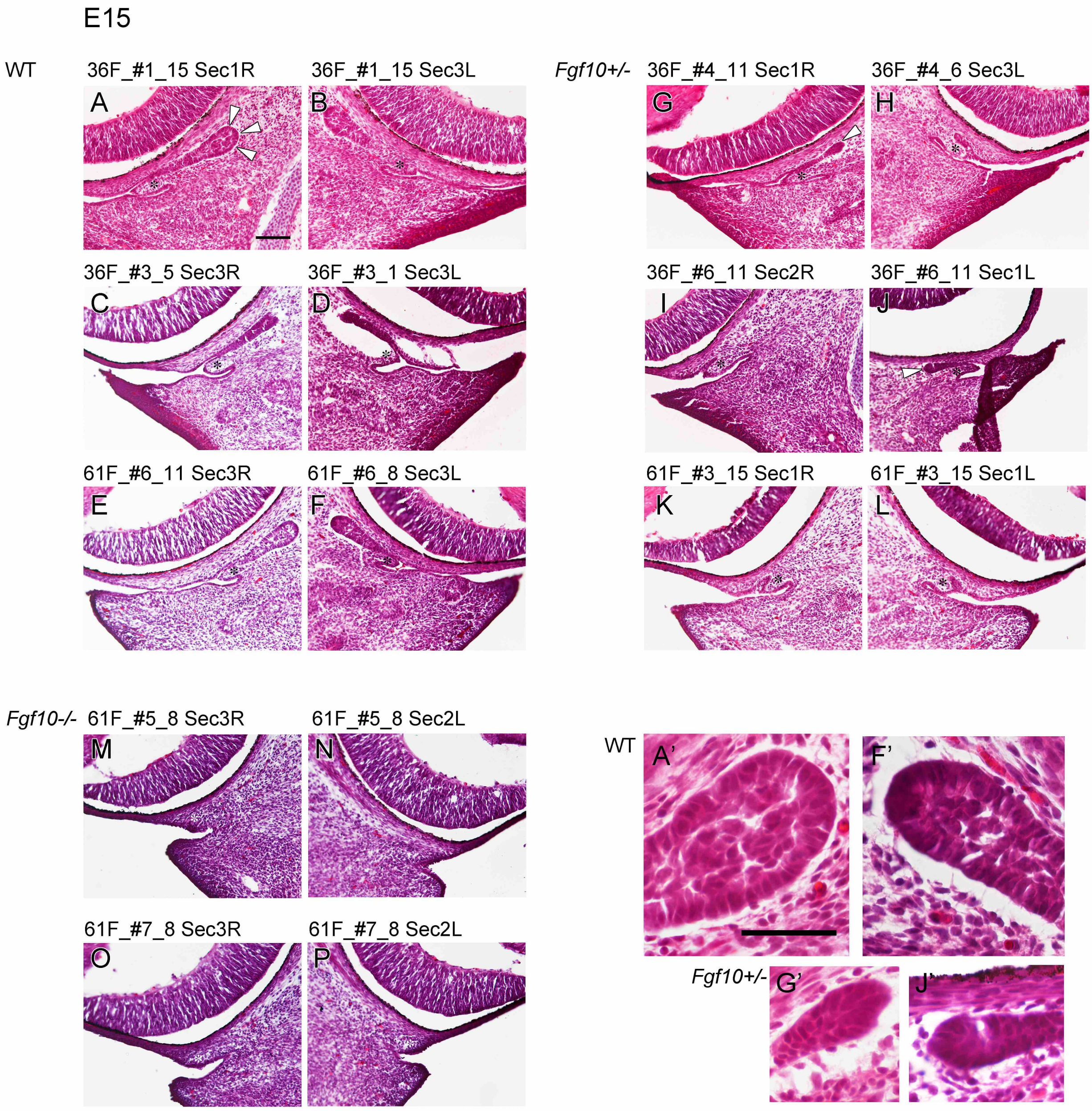

Figure S6. The early histology of invaginating surface ectoderm at E15, related to Fig. 2C′, D′. Arrowheads in (**A**) shows the tip of the invaginating epithelium along the eyeball, multilayered with columnar and cuboidal cells. Compare with the arrowhead in (**G**), indicating a smaller tip of the elongating glandular epithelium. The tips of the elongating glandular cells in (**A, F, G, J**) are enlarged in (**A′, F′, G′, J′**), respectively. Histology of *Fgf10*$^{-/-}$ samples on right (R) and left (L) sides are also shown in (**M-P**) (n = 2). Primordia for the nictitating membrane do not protrude well. Scale bars: 100 µm (**A-P**), 50 µm (**A′, F′, G′, J′**).

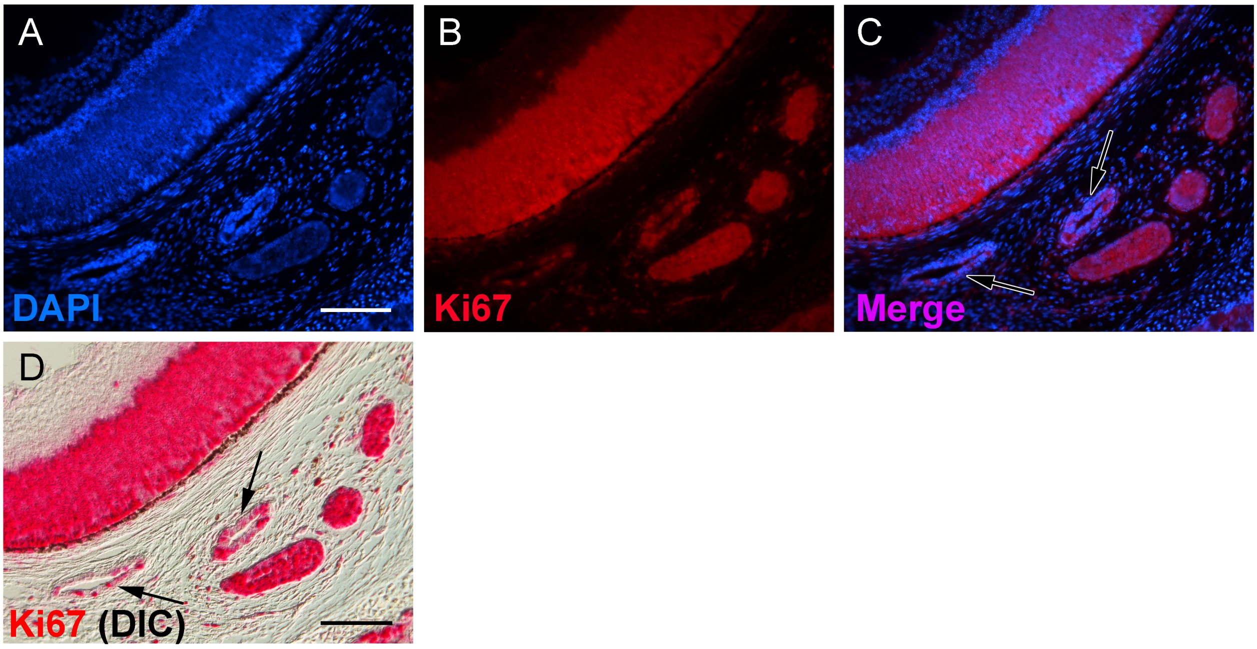

Figure S7. Ki67 expression is decreased in the cavitating HG primordia. Supporting data for Fig. 2F. Fluorescence microscopic images (**A-C**) and differential interference contrast image (**D**) of E19 mouse HGs. Localization of Ki67 protein is visualized with Vector Red. Arrows in (**C, D**) shows cavitating HG cells, in which fewer Ki67-positive cells are observed than in other three proliferating glandular portions. Scale bars: 100 μm (**A-C**), 100 μm (**D**).

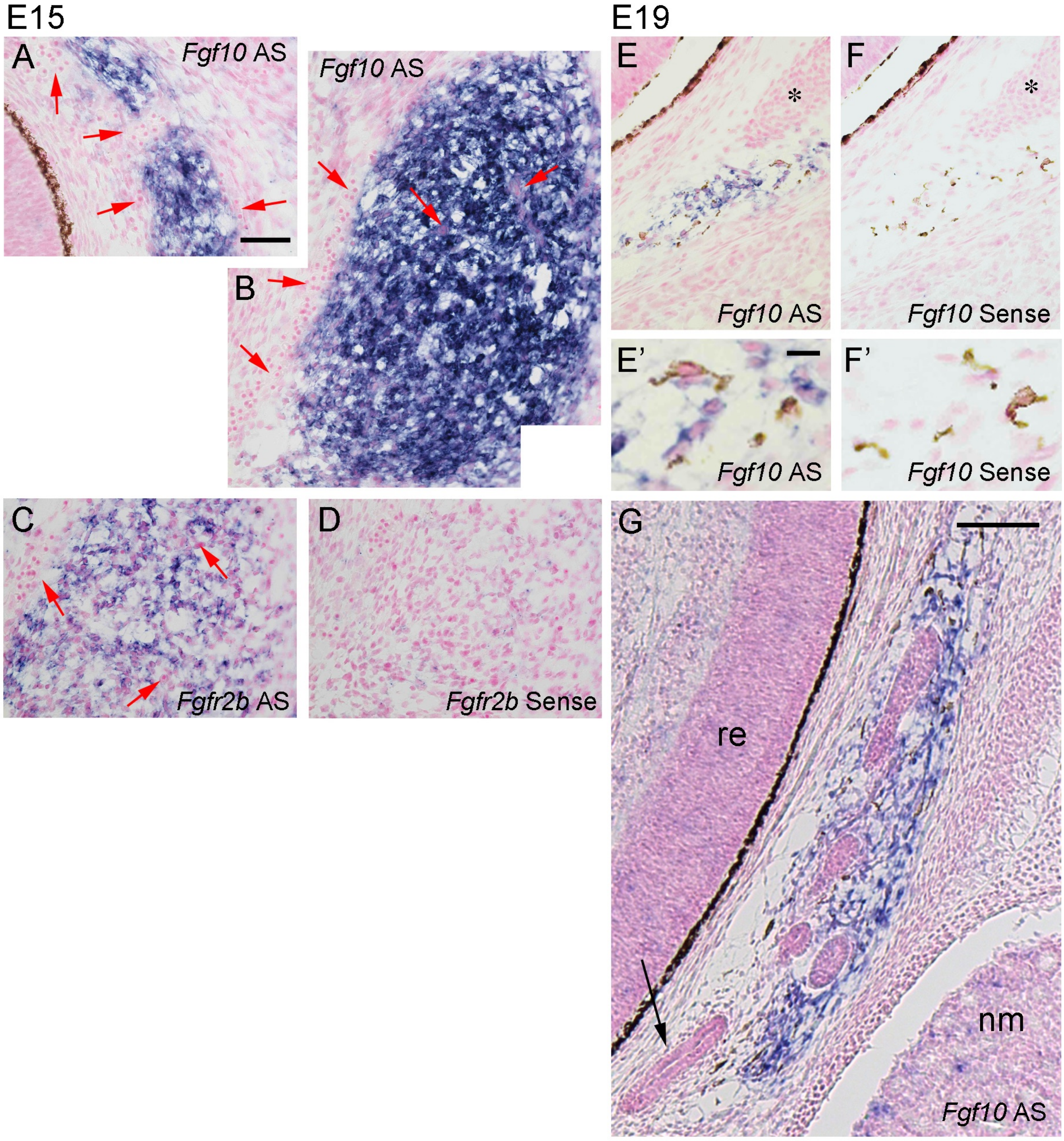

Figure S8. In situ hybridization of *Fgf10* (E15, E19) and *Fgfr2b* (E15) in the WT mouse head mesenchyme, related to Figures 3 and 4. (**A, B**) Two horizontal levels of the E15 WT head are shown, rostral (upper) (**A**) and caudal (lower) (**B**) levels. In panel (**A**), arrows show round cells abutting the *Fgf10*-expressing mesenchymal cells. In (**B**), arrows show the similar round cells, possibly hemangioblasts, abutting and intermingling with the *Fgf10*-expressing mesenchymal cells. (**C**) *Fgfr2b* expression in the *Fgf10*-expressing periocular mesenchymal domain. Arrows show the round cells without *Fgfr2b* expression, abutting and intermingling with the *Fgfr2b*-expressing mesenchymal cells. (**D**) Hybridized with an *Fgfr2b* sense probe as a negative control. (**E**) No *Fgf10* expression in the mesenchymal melanoblasts. (**F**) Hybridized with an *Fgf10* sense probe as a negative control. Asterisks in (**E, F**) show the developing cartilage core of the nictitating membrane. Magnified view of Harderian melanoblasts in (**E, F**) are shown in (**E′, F′**), respectively. (**G**) No *Fgf10* expression in the surrounding mesenchyme of HGs in tubulogenesis (arrow). Scale bars: 50 μm (**A-F**), 10 μm (**E′, F′**), 100 μm (**G**).

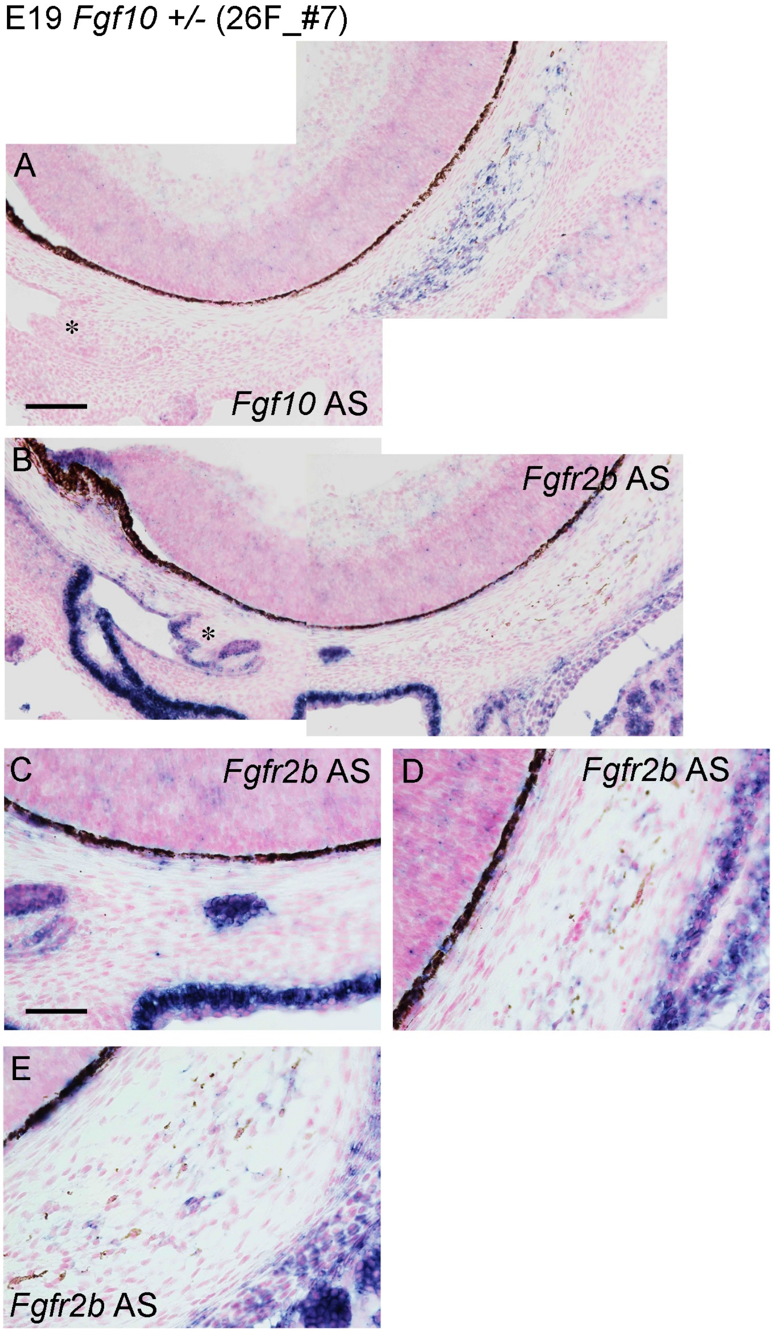

Figure S9. In situ hybridization of *Fgf10* and *Fgfr2b* in the E19 *Fgf10*$^{+/-}$ mouse HG primordium, related to Figure 4. Asterisks in (**A, B**) show the developing nictitating membrane. (**A**) High magnification of Fig. 4I. *Fgf10* is expressed in the HG mesenchyme not to contain developing glandular epithelial cells. (**B**) High magnification of Fig. 4K. *Fgfr2b* expression indicates that HG cells did not reach the HG mesenchyme. (**C**) High magnification of panel (**B**). Different expression levels of *Fgfr2b* are observed depending on the epithelia. (**D**) A more rostral (upper level) section of (**C**). A small population of cells without *Fgfr2b* expression is found in the HG mesenchyme. (**E**) A more caudal (lower level) section of panel (**C**). A few *Fgfr2b*-expressing cells are scattered in the HG mesenchyme. Scale bars: 100 μm (**A, B**), 50 μm (**C-E**).

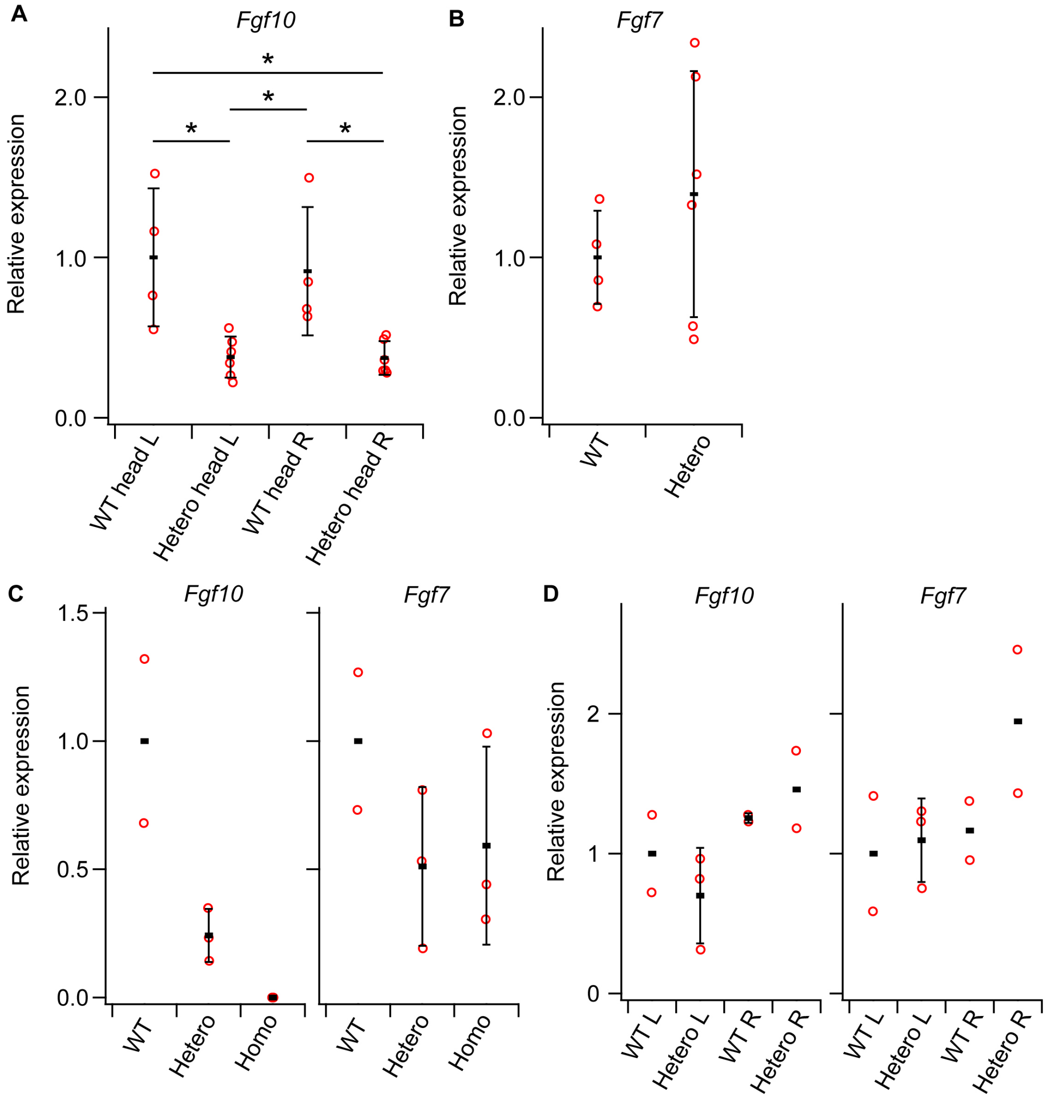

Figure S10. Relative expression levels of *Fgf10* and *Fgf7* in wild-type, *Fgf10*$^{+/-}$, and *Fgf10*$^{-/-}$ embryos and adult Harderian tissues as shown by qRT-PCR. Mean and standard deviation are indicated. Red plots show the individual data points. (**A**) Relative expression levels of *Fgf10* in wild-type (WT) and *Fgf10*$^{+/-}$ (Hetero) heads at E17.5. Total RNA was extracted, and cDNA were synthesized from left (L) and right (R) heads separately and processed for qPCR. n = 4 for WT and n = 6 for *Fgf10*$^{+/-}$ mice were used. (**B**) Relative expression levels of *Fgf7* in WT and *Fgf10*$^{+/-}$ heads at E17.5. n = 4 for WT, n = 6 for *Fgf10*$^{+/-}$ mice were used. (**C**) Relative expression levels of *Fgf10* and *Fgf7* in WT, *Fgf10*$^{+/-}$ and *Fgf10*$^{-/-}$ (Homo) embryos at E15. n= 2 for WT, n = 3 for *Fgf10*$^{+/-}$, and n = 3 for *Fgf10*$^{-/-}$ embryos were used. In the *Fgf10*+/- embryos, *Fgf10* expression tends to be decreased than wild-type. In the *Fgf10*-null embryos, *Fgf10* is not expressed at all. *Fgf7* expression levels did not tend to be upregulated in the *Fgf10*$^{+/-}$ or *Fgf10*-null embryos. (**D**) Relative expression levels of *Fgf10* and *Fgf7* in WT and *Fgf10*$^{+/-}$, right and left Harderian glands at 19 weeks. n = 2 for left WT, n = 3 for left *Fgf10*$^{+/-}$, n = 2 for right WT, and n = 2 for right *Fgf10*$^{+/-}$ HGs (as one right *Fgf10*$^{+/-}$ HG was degenerated). **P* < 0.05, nonparametric two-sided Wilcoxon rank-sum test with Benjamini–Hochberg correction.

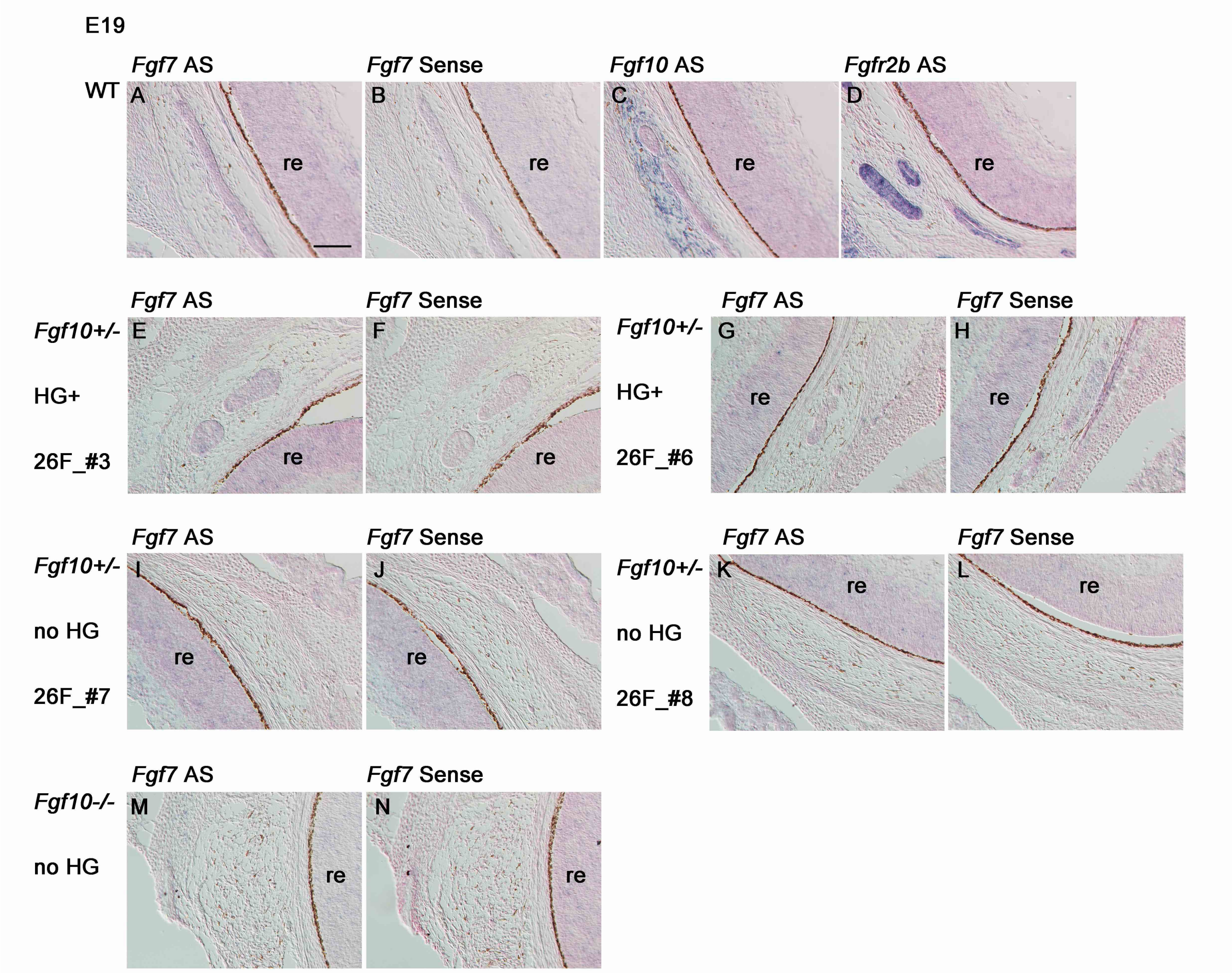

Figure S11. *Fgf7* is not expressed in the wild-type, *Fgf10*$^{+/-}$, or *Fgf10*$^{-/-}$ developing Harderian tissues at E19 as revealed by in situ hybridization. Expression of *Fgf10* and *Fgfr2b* is shown in (**C, D**) as experimental controls. High magnification and additional data for Fig. 4C-H. Scale bar: 100 µm (**A-N**).

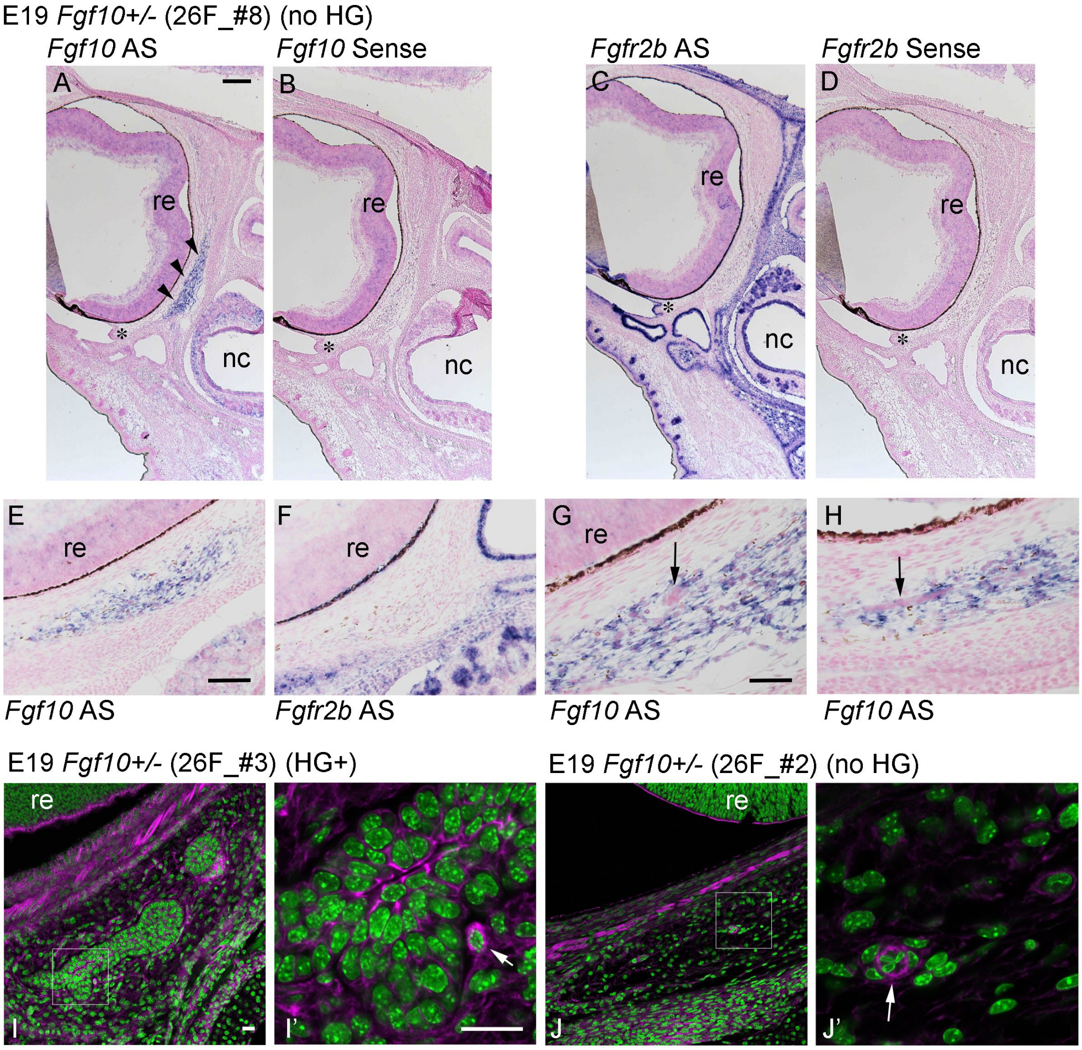

Figure S12. In situ hybridization of *Fgf10* and *Fgfr2b*, and localization of F-actin in other E19 *Fgf10*$^{+/-}$ HG primordia, related to Figure 4. Horizontal sections are shown. Nasal cavity (nc) is shown downward. In this *Fgf10*$^{+/-}$ embryo (26F_#8), there were no HG epithelial cells in the periocular mesenchyme, judging from *Fgfr2b* expression. (**A**) *Fgf10* is expressed in the HG mesenchyme without glandular epithelia. (**B**) Hybridized with an *Fgf10* sense probe as a negative control. (**C**) There are no *Fgfr2b*-expressing epithelial cells within the periocular mesenchyme. (**D**) Hybridized with an *Fgfr2b* sense probe as a negative control. (**E-H**) High magnification of the HG mesenchyme is shown. (**E**) A more caudal (lower level) section of panel (**A**). (**F**) A more rostral (upper level) section of (**C**). (**G**) High magnification of (**A**). Arrow shows round cells populated in the HG mesenchyme. (**H**) A more rostral (upper level) section of (**A**). Arrow shows thin tubular cells populated in the HG mesenchyme. (**I, I′, J, J′**) Confocal micrographs of frozen sections after phalloidin (magenta) and nuclear (green) stain. Boxed area in (**I, J**) are enlarged in (**I′, J′**), respectively. (**I′**) Localization of F-actin is relatively intense at the apical domain facing the HG lumen in the *Fgf10*$^{+/-}$ embryo (26F_#3) with developing HG. A cell in the HG mesenchyme (arrow) has relatively intense F-actin localization in the cytoplasm. (**J**) This *Fgf10*$^{+/-}$ embryo (26F_#2) has no HG development. In the HG mesenchyme, there is a round F-actin localization, enlarged in (**J′**). Cells (arrow in **J′**) are aligned in rosette formation. Localization of F-actin is also observed in the apical domain of the neural retina (re), vascular smooth muscles of the choroid, and developing extraocular muscles in (**I, J**). Scale bares: 2 μm (**A-D**), 100 μm (**E, F**), 50 μm (**G, H**), 20 μm (**I** and **J, I′** and **J′**).

Supplementary Tables

Table S1. **Phenotypes of Harderian glands in wild-type (WT) and *Fgf10+/-* mice at 74 weeks of age.**

Abbreviaitons: R, right; L, left; HG, Harderian gland; M, male; w, weeks; PFA, paraformaldehyde,

Definition: 3+, well developed; 2+ developed but less than wild-type male one; 1+ present but obviously small; absent, could not be found; degenerated, markedly small and blackened.

**Body weight (g)**	**Animal #**	**Genotype**	**Sex**	**Age**	**Fixation date**	**Fixative**	**R)HG**	**L)HG**
41	3M	WT	♂	74w	2022.10.20	FEKETE	3+	3+
44.2	4M	WT					3+	3+
49.5	7M	WT			2023.1.12	FEKETE	3+	3+
43.3	12M	WT			2023.8.9	4%PFA	3+	3+
35.8	13M	WT					3+	3+
37.6	14M	WT					3+	3+
26.4	2M	*Fgf10+/-*			2022.10.20	FEKETE	**degenerated**	3+
51.5	5M	*Fgf10+/-*					**degenerated**	3+
40.8	6M	*Fgf10+/-*			2023.1.12	FEKETE	3+	3+
43.4	9M	*Fgf10+/-*			2023.5.10	4%PFA	3+	3+
51.1	11M	*Fgf10+/-*					**degenerated**	3+
39.3	16M	*Fgf10+/-*			2023.10.12		3+	3+
54	2F	WT	♀	74w	2023.1.18	FEKETE	3+	3+
39.5	3F	WT			2023.5.10	4%PFA	3+	3+
41.1	10F	WT			2023.8.9		2+	2+
62.6	11F	WT			2023.10.12		3+	3+
55.2	14F	WT					3+	3+
35.5	4F	*Fgf10+/-*			2023.5.10		**degenerated**	3+
33.2	5F	*Fgf10+/-*					3+	**degenerated**
33	9F	*Fgf10+/-*					**degenerated**	3+
39.3	7F	*Fgf10+/-*			2023.8.9		**degenerated**	**degenerated**
51.9	12F	*Fgf10+/-*			2023.10.12		3+	3+
33	13F	*Fgf10+/-*					3+	3+

**Table S2.**	**Phenotypes of Harderian glands in wild-type (WT) and *Fgf10+/-* mice at postnatal day 18 (P18).**											
Abbreviaitons: R, right; L, left; HG, Harderian gland; PFA, paraformaldehyde.												
Definition: degenerated, markedly small and blackened.												
**Body weight (g)**	**Animal #**	**Genotype**	**Sex**	**Age**	**Fixation date**	**Fixative**	**R)HG (weight, mg)**		**R)HG weight / body weight**	**L)HG (weight, mg)**		**L)HG weight / body weight**
5.7	26F_1	WT	♂	P18	2023.1.6	4% PFA	3.1	developed	0.5439	3.5	developed	0.614
5.0	26F_2	WT					3.2	developed	0.64	2.7	developed	0.54
4.8	26F_3	WT					1.7	developed	0.3542	2.9	developed	0.6042
4.2	26F_6	WT	♀				3.5	developed	0.8333	3.6	developed	0.8571
2.5	26F_4	*Fgf10+/-*	♂				2.5	developed	1	1	**degenerated**	0.4
4.5	26F_5	*Fgf10+/-*					1.4	**degenerated**	0.3111	4.1	developed	0.9111
3.4	26F_7	*Fgf10+/-*	♀				3.4	developed	1	1.1	**degenerated**	0.3235
5.5	40F_1	WT	♂		2023.6.5		2.2	developed	0.4	2.8	developed	0.5091
7.4	40F_2	WT					4.4	developed	0.5946	4.3	developed	0.5811
7.9	40F_3	WT					4.6	developed	0.5823	4.5	developed	0.5696
6.8	40F_4	WT					3.8	developed	0.5588	3.4	developed	0.5
5.8	40F_5	*Fgf10+/-*					3.7	developed	0.6379	1	**degenerated**	0.1724
6.7	40F_6	*Fgf10+/-*					2.9	developed	0.4328	1.6	**degenerated**	0.2388
6.3	40F_7	*Fgf10+/-*					4.4	developed	0.6984	0.9	**degenerated**	0.1429
7.7	40F_8	*Fgf10+/-*					3.7	developed	0.4805	4	developed	0.5195

**Table S3.**	**Phenotypes of Harderian glands in wild-type (WT) and *Fgf10+/-* mice at early postnatal days (P0.5 to P14).**					
Abbreviaitons: R, right; L, left; HG, Harderian gland; PFA, paraformaldehyde.						
Definition: degenerated, markedly small and blackened.						
**Animal #**	**Genotype**	**Age**	**Fixation date**	**Fixative & treatment**	**R)HG**	**L)HG**
2	WT	P0.5	2022.12.15	4%PFA, decalcified	developed	developed
4	WT				developed	developed
6	WT				developed	developed
11	WT				developed	developed
1	*Fgf10+/-*				developed	**no HG**
3	*Fgf10+/-*				developed	developed
5	*Fgf10+/-*				developed	**no HG**
10	*Fgf10+/-*				developed	**no HG**
4	WT	P1	2022.3.31	4%PFA	developed	developed
6	WT				developed	developed
2	*Fgf10+/-*				**no HG**	**no HG**
8	*Fgf10+/-*				developed	developed
1	*Fgf10+/-*	P2.5	2023.9.8	4%PFA, decalcified	**delayed**	developed
2	WT			4%PFA, decalcified	developed	developed
3	WT			4%PFA, decalcified	developed	developed
4	WT			4%PFA, decalcified	developed	developed
5	*Fgf10+/-*			4%PFA, decalcified	developed	**delayed**
1	WT	P6	2023.9.1	4%PFA, decalcified	developed	developed
2	*Fgf10+/-*			4%PFA, decalcified	**degenerated**	developed
3	*Fgf10+/-*			4%PFA, decalcified	**degenerated**	developed
5	WT			4%PFA, decalcified	developed	developed
1	WT	P10	2023.3.29	4%PFA, decalcified	developed	developed
3	*Fgf10+/-*				**degenerated**	developed
1	WT	P14	2022.9.14	4%PFA	developed	developed
2	WT				developed	developed
4	*Fgf10+/-*				developed	**degenerated**
6	*Fgf10+/-*				developed	developed
2_1	WT		2022.11.18	FEKETE	developed	developed
2_2	WT				developed	developed
2_3	*Fgf10+/-*				**degenerated**	developed
2_4	WT				developed	developed

Table S4. Antibody and reagent list.

Antibodies	Dilution	Antigen retrieval	Company	Catalog #	IgG
anti-E-cadherin	50	boiling in citrate buffer (0.1M, pH6)	BD Bioscience	610181	mouse
anti-Pancytokeratin	200	boiling in citrate buffer (0.1M, pH6)	Abcam	ab86734	mouse
anti-Ki67	4500	boiling in citrate buffer (0.1M, pH6)	Abcam	ab15580	rabbit
anti-mouse IgG, Alexa Fluor 488	1000	N. A.	Thermo Fisher Scientific	A11029	goat
anti-rabbit IgG, Alexa Fluor 594	1000	N. A.	Thermo Fisher Scientific	A11037	goat

Reagents	Dilution	Concentration	Company	Catalog #
DAPI	5000	1mg/ mL	Dojindo	340-0791
Hoechst 33342	1000	1mg/ mL	Nacalai Tesque	04929-82
Rhodamine Phalloidin	50	N. A.	Thermo Fisher Scientific	R415
Mouse IgG isotype control	no dilution	0.5 ug/ mL	Thermo Fisher Scientific	08-6599
Normal rabbit IgG	11000	11 mg/ mL	Fujifilm Wako	148-09551
ImmPRESS Duet Double Staining HRP/AP Polymer Kit	N. A.	N. A.	Vector Laboratories	MP-7714
ImmPRESS-AP Horse Anti-Rabbit IgG Polymer Detection Kit	N. A.	N. A.	Vector Laboratories	MP-5401
DeadEnd™ Colorimetric TUNEL System	N. A.	N. A.	Promega	G7360
DeadEnd™ Fluorometric TUNEL System	N. A.	N. A.	Promega	G3250

N. A.: not applicable

Table S5. Oligonucleotide sequences for conventional ISH probes used in this study.

**Primers for cDNA cloning**				
**Primer name**	**Primer/DNA sequences (5'->3')**	**Gene name**	**Accession No.**	**Amplicon size (bp)**
mmu_Fgfr2_Fw	ATGGGATTACCGTCCACGTGGAGATATGGA	*Fgfr2*	NM_201601.2	2181
mmu_Fgfr2_Rv	TCATGTTTTAACACTGCCGTTTATGTGTGG			
mmu_Fgf10_Fw	CAACAAAACGCCAGCCGCCA	*Fgf10*	NM_008002.5	1279
mmu_Fgf10_Rv	CTATGTTTGGATCGTCATGGGGAGG			
mmu_Fgf7_Fw	AAGGTTAACAGTTTGGAAAGAGCGACGA	*Fgf7*	NM_008008.4	734
mmu_Fgf7_Rv	TTAGGTTATTGCCATAGGAAGAAAA			
**Primers for other purposes**				
**Primer name**	**Primer/DNA sequences (5'->3')**	**Usage**		
T7 primer	GTAATACGACTCACTATAGGG	Sequencing cDNAs inserted in pBluescriptII KS+ and preparation of riboprobe synthesis template		
T3 primer	AATTAACCCTCACTAAAGGAA	Sequencing cDNAs inserted in pBluescriptII KS+ and preparation of riboprobe synthesis template		
under_T7prom_pBSIIKSplus	CGCTCTAGAACTAGTGGATC	Preparation of riboprobe synthesis template for Fgf10 and Fgf7		
under_T3prom_pBSIIKSplus	AGGTCGACGGTATCGATAAG	Preparation of riboprobe synthesis template for Fgf10 and Fgf7		
SP6_under_T7prom_pBSIIKSplus	CATTTAGGTGACACTATAGAACGCTCTAGAACTAGTG[illegible]	Preparation of riboprobe synthesis template for Fgf10 and Fgf7		
SP6_under_T3prom_pBSIIKSplus	CATTTAGGTGACACTATAGAAAGGTCGACGGTATCG ATAAGC	Preparation of riboprobe synthesis template for Fgf10 and Fgf7		
Fgfr2_seq_Fw	TGCAAGGTTTACAGCGATGC	Sequencing isoform specific region of Fgfr2b cDNA clones		

Table S6. Oligonucleotide sequences and other information for SABER-FISH in this study.			
Hairpins and branches			
**Oligo name**	**Nucleotide sequence (5'->3')**	**Usage**	**Reference**
h.67.31.tail	ATTATTCACTGGGCCTTTTGGCCCAGTGAATAATATTATGTGATTTTTTT	Remap primer sequence in probe from no.67 to no.31	Kishi et al. 2019
h.31.31.tail	ATTATTCACTGGGCCTTTTGGCCCAGTGAATAATAGTGAATAATTTTTTT	Extension of probe or branch oligos	Kishi et al. 2019
h.27.27.tail	ACATCATCATGGGCCTTTTGGCCCATGATGATGTATGATGATGTTTTTTT	Extension of probe or branch oligos	Kishi et al. 2019
h.28.28.tail	ACAACTTAACGGGCCTTTTGGCCCGTTAAGTTGTGTTAAGTTGTTTTTTT	Extension of probe or branch oligos	Kishi et al. 2019
h.60.60.tail	AACTAACTATGGGCCTTTTGGCCCATAGTTAGTTATAGTTAGTTTTTTTT	Extension of probe or branch oligos	Kishi et al. 2019
h.73.73.tail	ATTCCTAATCGGGCCTTTTGGCCCGATTAGGAATGATTAGGAATTTTTTT	Extension of probe or branch oligos	Kishi et al. 2019
31*.31*.31*.31*.27	AGTGAATAATAGTGAATAATAGTGAATAATAGTGAATAATTTCATCATCAT	branch for signal amplification in SABER	Kishi et al. 2019
27*.27*.27*.28	ATGATGATGTATGATGATGTATGATGATGTTTCAACTTAAC	branch for signal amplification in SABER	Kishi et al. 2019
60*.60*.60*.60*.73	ATAGTTAGTTATAGTTAGTTATAGTTAGTTATAGTTAGTTTTTTCCTAATC	branch for signal amplification in SABER	Kishi et al. 2019
28*.488	/5ATTO488N/TTGTTAAGTTGTGTTAAGTTGT	Fluorescent detection	Kishi et al. 2019
73*.565	/5ATTO565N/TTGATTAGGAATGATTAGGAAT	Fluorescent detection	Kishi et al. 2019
Clean.G	CCCCGAAAGTGGCCTCGGGCCTTTTGGCCCGAGGCCACTTTCG	Removal of dGTP contaminated in primer exchange reaction	Kishi et al. 2019

**Pool name**	**Sequence**
mmu_Fgf10_5utr_cds.67	TGTGAAGTCTTCCGAAAGCCACTTCTTGTTTGGGGtttTCACATAAT
mmu_Fgf10_5utr_cds.67	GTCTCTTTGGAGTTGTCAGAACTGGAGGCAGCTGCtttTCACATAAT
mmu_Fgf10_5utr_cds.67	TCTCTGCAGAGTCCCAGCCTGCTGGCCACTTAAAAtttTCACATAAT
mmu_Fgf10_5utr_cds.67	CAGGACGGTGAACAAAACCCAAAGTGGGTGGGGTGtttTCACATAAT
mmu_Fgf10_5utr_cds.67	ACTCCTTTCTTCACCATGTTAGATGCAAAAGAGGTCTGAAAtttTCACATAAT
mmu_Fgf10_5utr_cds.67	GAACTGGTGGTGTCGGTCACCAGAGCTCTTCGCTCtttTCACATAAT
mmu_Fgf10_5utr_cds.67	TAACTTCTAGGAAGGACCGGCTGCCTGTCCTCGCTCtttTCACATAAT
mmu_Fgf10_5utr_cds.67	TACGGATCTGGCCAGAAGCGAGTGCACCAACATCCtttTCACATAAT
mmu_Fgf10_5utr_cds.67	ACATACTGGAAGGGTAAGACCTGCTGCGAGGCAGAtttTCACATAAT
mmu_Fgf10_5utr_cds.67	AACTCTCGGCACTGGAAATTGTCTCATCAGAAGGAtttTCACATAAT
mmu_Fgf10_5utr_cds.67	AGGCACAATGTGTCAGTATCCATTTCCACATTGTACTGAtttTCACATAAT
mmu_Fgf10_5utr_cds.67	AGCTTGGCAGGTGACAGGGAACGAAGACACCAAAAtttTCACATAAT
mmu_Fgf10_5utr_cds.67	CTTGGAGGTGATTGTAGCTCCGCACATGCCTTCCCtttTCACATAAT
mmu_Fgf10_5utr_cds.67	GGTGAAGGAGAACAGCCTTCTCCAGCGGACATCTCtttTCACATAAT
mmu_Fgf10_5utr_cds.67	TGACCTTGCCGTTCTTCTCAATCGTGAGAAAGTACTTtttTCACATAAT
mmu_Fgf10_5utr_cds.67	CTGTTGATGGCTTTGACGGCAACAACTCCGATTTCtttTCACATAAT
mmu_Fgf10_5utr_cds.67	TGAGCCATAGAGTTTCCCCTTCTTGTTCATGGCTAAGTAATtttTCACATAAT
mmu_Fgf10_5utr_cds.67	GCTGCCAGTTAAAAGATGCATAGGTGTTGTATCCATTTTCCtttTCACATAAT
mmu_Fgf10_5utr_cds.67	TCCATTCAATGCCACATACATTTGCCTGCCATTGTtttTCACATAAT
mmu_Fgf10_5utr_cds.67	GATCGTCATGGGGAGGAAGTGAGCAGAGGTGTTTTtttTCACATAAT
mmu_Fgf10_5utr_cds.67	GGTTGTACTGCATCCACCAACAGTGTTTTCTTCTATGTTTGtttTCACATAAT

Pool name	Sequence
mmu_Fgfr2b.60	GTAATCCCATCTGCACACTTCTTCTGAGACCATGGGtttACTAACTAT
mmu_Fgfr2b.60	CCTGGTCCTCTTCCATATCTCCACGTGGACGtttACTAACTAT
mmu_Fgfr2b.60	CCCCAGCTGACCATGGTCACAGTGCCAATCtttACTAACTAT
mmu_Fgfr2b.60	CAGGGACAAGGTTGCCATGGTGACCAAGACtttACTAACTAT
mmu_Fgfr2b.60	TATCCTCAACTAAACTGAAGGAGGGCCGGGCtttACTAACTAT
mmu_Fgfr2b.60	CAGGGACAGCGTGGAGCCGCTTCTCCATCTtttACTAACTAT
mmu_Fgfr2b.60	CCCAGCCGGACAGCGGAACTTCACAGTGTTtttACTAACTAT
mmu_Fgfr2b.60	TTTTAACCACCTCATTGTGGGCGTTGGATTCCCtttACTAACTAT
mmu_Fgfr2b.60	CAATGCGATGCTCCTGCTTAAACTCCTTCCCGtttACTAACTAT
mmu_Fgfr2b.60	GACCACACTTTCCATAATAAGGCTCCAGTGCTGGTtttACTAACTAT
mmu_Fgfr2b.60	CAGGCAGGTGTAGTTGCCTTTGTCTGACGGtttACTAACTAT
mmu_Fgfr2b.60	TAGGTGTGGTTGATGGACCCGTATTCATTCTCCACtttACTAACTAT
mmu_Fgfr2b.60	ACCGTGGAGGCATTTGCAGGCAGTCCAGCTTtttACTAACTAT
mmu_Fgfr2b.60	TTGCAGACAAACTCCACATCCCCTCCGACCtttACTAACTAT
mmu_Fgfr2b.60	TGGATGTGGGGCTGGGCATCGCTGTAAACCtttACTAACTAT
mmu_Fgfr2b.60	TACTGCCGTTCTTTTCCACGTGCTTGATCCACtttACTAACTAT
mmu_Fgfr2b.60	TGAGGTAGGGCAGCCCATCAGGCCCGTATTtttACTAACTAT
mmu_Fgfr2b.60	AGCCAGCACTTCTGCATTGGAGCTATTTATCCCCtttACTAACTAT
mmu_Fgfr2b.60	TATATTCCCCAGCATCCATCTCCGTCACATTGAACAGtttACTAACTAT
mmu_Fgfr2b.60	GTGAGCCAGGCAGACTGGTTGGCCTGCCCTATATtttACTAACTAT
mmu_Fgfr2b.60	ATAGCTATCTCCAGATAATCTGGGGAAGCCGTGATCTtttACTAACTAT
mmu_Fgfr2b.60	ACCATGCAGGCGATTAAGAAGACCCCTATGCAtttACTAACTAT
mmu_Fgfr2b.60	CGTGGTCTTCATTCGGCAAAAGATGACTGTCACCtttACTAACTAT
mmu_Fgfr2b.60	TGGCTGGCTGCTGAAGTCTGGCTTCTTGGTtttACTAACTAT
mmu_Fgfr2b.60	GGGGATGCGCTTGGTCAGCTTGTGCACAGCtttACTAACTAT
mmu_Fgfr2b.60	TTATCCTCACCAGCGGGGTGTTGGAGTTCAtttACTAACTAT
mmu_Fgfr2b.60	GGGTGTCCGCTGTTGAGGACAGACGCGTTGtttACTAACTAT
mmu_Fgfr2b.60	CAACTCATACTCGGAGACCCCTGCTAGCATCGtttACTAACTAT
mmu_Fgfr2b.60	GGTTTGTCTTTATCGATTCCCACTGCTTCAGCCATGtttACTAACTAT
mmu_Fgfr2b.60	ATCTTCACTGCCACGGTGACCGCCTCCTTGtttACTAACTAT
mmu_Fgfr2b.60	TCCATCTCTGATACCAGATCAGACAGGTCCTTCTCTGtttACTAACTAT
mmu_Fgfr2b.60	GGGCTCGGAGGTATTCCCGGAGGTTGCCTTtttACTAACTAT
mmu_Fgfr2b.60	ATGTCATAGGAGTACTCCATGCCAGGTGGCCtttACTAACTAT
mmu_Fgfr2b.60	AAGGTCATCTGCTCCTCGGGGACACGGTTAtttACTAACTAT
mmu_Fgfr2b.60	AGCTGGTAGGTGCAGGACACCAAGTCCTTGtttACTAACTAT
mmu_Fgfr2b.60	TTTTGGGAAGCCAAGTACTCCATGCCTCTAGCCtttACTAACTAT
mmu_Fgfr2b.60	TCACATTGTTTTCTGTTACCAACACGTTTCTGGCAGCtttACTAACTAT
mmu_Fgfr2b.60	ATAGTAGTCTATGTTGTTGATATCCCTGGCCAGGCCAtttACTAACTAT
mmu_Fgfr2b.60	AAAAGGGCTTCAGGAGCCATCCACTTGACTGtttACTAACTAT
mmu_Fgfr2b.60	CCCCTAAAGTAAAGATCTCCCACATTAACACCCCGAAtttACTAACTAT
mmu_Fgfr2b.60	AAAAGTTCCTCCACGGGAATCCCTGGGTAGGtttACTAACTAT
mmu_Fgfr2b.60	AGTTGGTGGGCTTGTCCATCCTGTGTCCCTtttACTAACTAT
mmu_Fgfr2b.60	CAACTGCTTGAATGTGGGTCTCTGTGAGGGTACAtttACTAACTAT
mmu_Fgfr2b.60	TGGTTGTGAGAGTCAGAATTCGATCCAAGTCTTCGACtttACTAACTAT
mmu_Fgfr2b.60	TGTGTCGGGGTAACTAGGAGAATACTGTTCGAGAGGtttACTAACTAT
mmu_Fgfr2b.60	ACAGAATCGTCCCCTGAAGAACAAGAGCTCCTtttACTAACTAT
mmu_Fgfr2b.60	CAGGGTTCATAAGGCATGGGGTCTGGAGAAAACtttACTAACTAT
mmu_Fgfr2b.60	TTTAACACTGCCGTTTATGTGTGGATACTGAGGCAGAtttACTAACTAT

Remapping information in PER reaction				
Primer sequence of mmu_Fgf10_5utr_cds.67 was remapped to no.31.				
Primer sequences of mmu_Fgfr2b.60 and branches were not remapped.				
**Applied probes and branches in each hybridization step**				
Target genes	probe hyb	1st branch hyb	2nd branch hyb	Fluorescent oligo hyb
Fgf10 & *Fgfr2b*	mmu_Fgf10_5utr_cds.31 (extended)	31*.31*.31*.31*.27 (extendec	27*.27*.27*.28 (extended	28*.488
	mmu_Fgfr2b.60 (extended)	60*.60*.60*.60*.73 (extendec	not applicable	73*.565

Table S7. qPCR primers used in this study.				
**Gene name**	**Forward (5'-3')**	**Reverse (5'-3')**	**Amplicon size (bp)**	**Reference**
Fgf10	CGGGACCAAGAATGAAGACT	GCAACAACTCCGATTTCCAC	69	Finburgh et al., 2023, Invest Opthal Vis Sci
Fgf7	ATAGAAACAGGTCGTGACAAGG	CAGACAGCAGACACGGAAC	105	Du et al. 2016, Gene Exp Patterns
Gapdh	AGGTTGTCTCCTGCGACTTCA	TGGTCCAGGGTTTCTTACTCC	294	Liu et al., 2008, Biochem J

References

1. Du, W.; Prochazka, J.; Prochazkova, M.; Klein, O.D. Expression of FGFs during early mouse tongue development. *Gene Expr Patterns* **2016,** 20, 81-87, DOI:10.1016/j.gep.2015.12.003.
2. Finburgh, E.N.; Mauduit, O.; Noguchi, T.; Bu, J.J.; Abbas, A.A.; Hakim, D.F.; Bellusci, S.; Meech, R.; Makarenkova, H.P.; Afshari, N.A. Role of FGF10/FGFR2b Signaling in Homeostasis and Regeneration of Adult Lacrimal Gland and Corneal Epithelium Proliferation. *Invest Ophthalmol Vis Sci* **2023,** 64, 21, DOI:10.1167/iovs.64.1.21.
3. Liu, X.; Li, P.; Liu, P.; Xiong, R.; Zhang, E.; Chen, X.; Gu, D.; Zhao, Y.; Wang, Z.; Zhou, Y. The essential role for c-Ski in mediating TGF-beta1-induced bi-directional effects on skin fibroblast proliferation through a feedback loop. *Biochem J* **2008,** 409, 289-297, DOI:10.1042/BJ20070545.

Table S8. Source data for qPCR.

For Figure S10A:

Sample	Target gene	Cq	ΔCq	$2^{-\Delta Cq}$	$2^{-\Delta\Delta Cq}$	Group	mean $2^{-\Delta Cq}$	sdev $2^{-\Delta Cq}$	mean $2^{-\Delta\Delta Cq}$	sdev $2^{-\Delta\Delta Cq}$
sample no.2 WT head L	Gapdh	17.9816								
sample no.5 WT head L	Gapdh	16.9181								
sample no.7 WT head L	Gapdh	16.9352								
sample no.10 WT head L	Gapdh	19.1147								
sample no.2 WT head R	Gapdh	17.5064								
sample no.5 WT head R	Gapdh	17.5469								
sample no.7 WT head R	Gapdh	15.975								
sample no.10 WT head R	Gapdh	18.6673								
sample no.1 Hetero head L	Gapdh	16.8165								
sample no.3 Hetero head L	Gapdh	17.048								
sample no.4 Hetero head L	Gapdh	16.2154								
sample no.6 Hetero head L	Gapdh	16.1016								
sample no.8 Hetero head L	Gapdh	18.135								
sample no.9 Hetero head L	Gapdh	16.8556								
sample no.1 Hetero head R	Gapdh	17.5669								
sample no.3 Hetero head R	Gapdh	17.1665								
sample no.4 Hetero head R	Gapdh	17.3831								
sample no.6 Hetero head R	Gapdh	17.8526								
sample no.8 Hetero head R	Gapdh	17.3779								
sample no.9 Hetero head R	Gapdh	16.7449								
sample no.2 WT head L	Fgf10	31.5277	13.5461	8.36004E-05	0.551624549	WT head L	0.0001516	6.53317E-05	1	0.431082
sample no.5 WT head L	Fgf10	29.9971	13.0789	0.00011557	0.762570713					
sample no.7 WT head L	Fgf10	29.4055	12.4704	0.000176217	1.16274223					
sample no.10 WT head L	Fgf10	31.1956	12.0809	0.000230825	1.523064119					
sample no.2 WT head R	Fgf10	30.8575	13.351	9.57056E-05	0.631498984	WT head R	0.0001385	6.05963E-05	0.914032	0.399836
sample no.5 WT head R	Fgf10	30.7921	13.2452	0.000102991	0.679571657					
sample no.7 WT head R	Fgf10	28.9014	12.9264	0.000128455	0.847591179					
sample no.10 WT head R	Fgf10	30.7726	12.1054	0.000226945	1.497466189					
sample no.1 Hetero head L	Fgf10	31.4179	14.6014	4.02296E-05	0.265448786	Hetero head L	5.739E-05	1.94029E-05	0.378712	0.128027
sample no.3 Hetero head L	Fgf10	31.2874	14.2394	5.1704E-05	0.341161506					
sample no.4 Hetero head L	Fgf10	30.1839	13.9685	6.23808E-05	0.411610779					
sample no.6 Hetero head L	Fgf10	29.868	13.7664	7.17637E-05	0.473521901					
sample no.8 Hetero head L	Fgf10	31.6603	13.5253	8.48154E-05	0.559642006					
sample no.9 Hetero head L	Fgf10	31.7221	14.8665	3.34759E-05	0.220885778					
sample no.1 Hetero head R	Fgf10	31.7217	14.1549	5.48231E-05	0.361742424	Hetero head R	5.657E-05	1.59469E-05	0.373254	0.105223
sample no.3 Hetero head R	Fgf10	30.8813	13.7148	7.43743E-05	0.490748067					
sample no.4 Hetero head R	Fgf10	31.8227	14.4396	4.5002E-05	0.296939336					
sample no.6 Hetero head R	Fgf10	31.4934	13.6408	7.82911E-05	0.516592305					
sample no.8 Hetero head R	Fgf10	31.8352	14.4573	4.44552E-05	0.2933308					
sample no.9 Hetero head R	Fgf10	31.2684	14.5235	4.24606E-05	0.280169825					

Table S8. Source data for qPCR.

For Figure S10B:

Sample	Target gene	Cq	ΔCq	$2^{-\Delta Cq}$	$2^{-\Delta\Delta Cq}$	Group	mean $2^{-\Delta Cq}$	sdev $2^{-\Delta Cq}$	mean $2^{-\Delta\Delta Cq}$	sdev $2^{-\Delta\Delta Cq}$
sample no.2 WT head	Gapdh	16.7813								
sample no.5 WT head	Gapdh	20.9939								
sample no.7 WT head	Gapdh	17.2176								
sample no.10 WT head	Gapdh	18.7568								
sample no.1 Hetero head	Gapdh	17.1271								
sample no.3 Hetero head	Gapdh	16.9754								
sample no.4 Hetero head	Gapdh	18.1147								
sample no.6 Hetero head	Gapdh	19.1906								
sample no.8 Hetero head	Gapdh	18.1532								
sample no.9 Hetero head	Gapdh	18.9948								
sample no.2 WT head	Fgf7	29.0172	12.2359	0.00021	0.6938261	WT head	0.0002988	8.70E-05	1	0.291145
sample no.5 WT head	Fgf7	32.588	11.5941	0.00032	1.082529					
sample no.7 WT head	Fgf7	29.1469	11.9293	0.00026	0.8581177					
sample no.10 WT head	Fgf7	30.0159	11.2591	0.00041	1.3655306					
sample no.1 Hetero head	Fgf7	29.8681	12.741	0.00015	0.4888723	Hetero head	0.00041704	0.0002294	1.39569	0.767629
sample no.3 Hetero head	Fgf7	29.49	12.5147	0.00017	0.5719067					
sample no.4 Hetero head	Fgf7	28.5974	10.4827	0.0007	2.3389245					
sample no.6 Hetero head	Fgf7	29.8099	10.6193	0.00064	2.1275485					
sample no.8 Hetero head	Fgf7	29.4532	11.3	0.0004	1.3273015					
sample no.9 Hetero head	Fgf7	30.0996	11.1048	0.00045	1.5195914					

Table S8. Source data for qPCR.

For Figure S10C:

Sample	Target gene	Cq	Cq Average (replicate)	ΔCq	$2^{-\Delta Cq}$	$2^{-\Delta\Delta Cq}$	Group	mean $2^{-\Delta Cq}$	sdev $2^{-\Delta Cq}$	mean $2^{-\Delta\Delta Cq}$	sdev $2^{-\Delta\Delta Cq}$
61F-E15 Body WT No.2	Gapdh	28.6745	28.652070								
61F-E15 Body WT No.2	Gapdh	28.6296									
61F-E15 WT No.6	Gapdh	29.1984	29.164865								
61F-E15 WT No.6	Gapdh	29.1314									
61F-E15 Hetero No.1	Gapdh	29.3538	29.27403049								
61F-E15 Hetero No.1	Gapdh	29.19426									
61F-E15 Body Hetero No.3	Gapdh	27.4223	27.423028								
61F-E15 Body Hetero No.3	Gapdh	27.4237									
61F-E15 Hetero No.8	Gapdh	27.9493	27.810529								
61F-E15 Hetero No.8	Gapdh	27.6718									
61F-E15 Body Homo No.4	Gapdh	28.3464	28.408907								
61F-E15 Body Homo No.4	Gapdh	28.4714									
61F-E15 Homo No.5	Gapdh	27.6902	27.818714								
61F-E15 Homo No.5	Gapdh	27.9472									
61F-E15 Homo No. 7	Gapdh	28.7818	28.942924								
61F-E15 Homo No. 7	Gapdh	29.1040									
61F-E15 Body WT No.2	Fgf10	34.2111	33.897653	5.245582	0.026359		WT	0.019969	0.0090368	1	0.45254244
61F-E15 Body WT No.2	Fgf10	33.5842									
61F-E15 WT No.6	Fgf10	36.4497	35.367307	6.202442	0.013579						
61F-E15 WT No.6	Fgf10	34.2849									
61F-E15 Hetero No.1	Fgf10		36.15440863	7.74550144	0.004659848		Hetero	0.00482528	0.002063	0.24163854	0.10330913
61F-E15 Hetero No.1	Fgf10	36.15441									
61F-E15 Body Hetero No.3	Fgf10	35.3712	34.588402	7.165374	0.006966						
61F-E15 Body Hetero No.3	Fgf10	33.8056									
61F-E15 Hetero No.8	Fgf10	36.3215	36.265526	8.454997	0.002850						
61F-E15 Hetero No.8	Fgf10	36.2096									
61F-E15 Body Homo No.4	Fgf10		#DIV/0!	#DIV/0!	0.000000		Homo	0	0	0	0
61F-E15 Body Homo No.4	Fgf10										
61F-E15 Homo No.5	Fgf10		#DIV/0!	#DIV/0!	0.000000						
61F-E15 Homo No.5	Fgf10										
61F-E15 Homo No. 7	Fgf10		#DIV/0!	#DIV/0!	0.000000						
61F-E15 Homo No. 7	Fgf10										
61F-E15 Body WT No.2	Fgf7	32.9951	32.931392	4.279322	0.051499		WT	0.0406055	0.0154057	1	0.37939934
61F-E15 Body WT No.2	Fgf7	32.8676									
61F-E15 WT No.6	Fgf7	33.7701	34.237699	5.072834	0.029712						
61F-E15 WT No.6	Fgf7	34.7053									
61F-E15 Hetero No.1	Fgf7	33.82358	34.20165948	4.92762899	0.032857602		Hetero	0.0207432	0.0125548	0.510847053	0.30918964
61F-E15 Hetero No.1	Fgf7	34.57974									
61F-E15 Body Hetero No.3	Fgf7	32.5120	32.957074	5.534046	0.021582						
61F-E15 Body Hetero No.3	Fgf7	33.4022									
61F-E15 Hetero No.8	Fgf7	33.9730	34.814768	7.004239	0.007790						
61F-E15 Hetero No.8	Fgf7	35.6565									
61F-E15 Body Homo No.4	Fgf7	33.6443	34.212817	5.803909	0.017900		Homo	0.0240427	0.015663	0.592104518	0.38573592
61F-E15 Body Homo No.4	Fgf7	34.7814									
61F-E15 Homo No.5	Fgf7	33.7874	34.154345	6.335631	0.012382						
61F-E15 Homo No.5	Fgf7	34.5213									
61F-E15 Homo No. 7	Fgf7	33.2714	33.521686	4.578761	0.041846						
61F-E15 Homo No. 7	Fgf7	33.7719									

Table S8. Source data for qPCR.

For Figure S10D:

Sample	Target gene	Cq	Cq Average	ΔCq	$2^{-\Delta Cq}$	$2^{-\Delta\Delta Cq}$	Group	mean $2^{-\Delta Cq}$	sdev $2^{-\Delta Cq}$	mean $2^{-\Delta\Delta Cq}$	sdev $2^{-\Delta\Delta Cq}$
53M WT HG-L	Gapdh	16.10203	16.12702846								
53M WT HG-L	Gapdh	16.15202									
54M WT HG-L	Gapdh	16.14256	16.12208878								
54M WT HG-L	Gapdh	16.10162									
53M WT HG-R	Gapdh	14.85334	14.83144972								
53M WT HG-R	Gapdh	14.80956									
54M WT HG-R	Gapdh	15.09131	15.08992328								
54M WT HG-R	Gapdh	15.08854									
55M Hetero HG-L	Gapdh	17.38153	17.36034165								
55M Hetero HG-L	Gapdh	17.33916									
56M Hetero HG-L	Gapdh	16.21122	16.16391459								
56M Hetero HG-L	Gapdh	16.11661									
57M Hetero HG-L	Gapdh	17.49815	17.43100231								
57M Hetero HG-L	Gapdh	17.36385									
55M Hetero HG-R	Gapdh	29.58962	29.54168417								
55M Hetero HG-R	Gapdh	29.49374									
56M Hetero HG-R	Gapdh	15.19625	14.92362845								
56M Hetero HG-R	Gapdh	14.65101									
57M Hetero HG-R 2	Gapdh	15.10519	15.05497347								
57M Hetero HG-R 2	Gapdh	15.00475									
53M WT HG-L	Fgf10	30.1899	30.33717334	14.21014488	5.2762E-05	1.277108	WT L	0.0000415	1.62635E-05	1	0.391892
53M WT HG-L	Fgf10	30.48445									
54M WT HG-L	Fgf10	31.42341	31.16120553	15.03911676	2.9701E-05	0.722892					
54M WT HG-L	Fgf10	30.89901									
53M WT HG-R	Fgf10	29.11254	29.08217958	14.25072987	5.1298E-05	1.228916	WT R	5.20E-05	1.41E-06	1.253012	0.034077
53M WT HG-R	Fgf10	29.05182									
54M WT HG-R	Fgf10	29.30367	29.2925129	14.20258962	5.3039E-05	1.277108					
54M WT HG-R	Fgf10	29.28136									
55M Hetero HG-L	Fgf10	32.10615	31.97800392	14.61766227	3.9778E-05	0.963855	Hetero L	2.90E-05	1.42E-05	0.698795	0.341624
55M Hetero HG-L	Fgf10	31.84986									
56M Hetero HG-L	Fgf10	31.07703	30.99827238	14.83435779	3.4231E-05	0.819277					
56M Hetero HG-L	Fgf10	30.91952									
57M Hetero HG-L	Fgf10	33.41893	33.67474088	16.24373857	1.2887E-05	0.313253					
57M Hetero HG-L	Fgf10	33.93056									
55M Hetero HG-R	Fgf10	36.57081	36.97201124	7.430327068	0.00579761						
55M Hetero HG-R	Fgf10	37.37322									
56M Hetero HG-R	Fgf10	29.15814	29.236888	14.31325955	4.9122E-05	1.180723	Hetero R	6.05E-05	1.63E-05	1.457831	0.391892
56M Hetero HG-R	Fgf10	29.31563									
57M Hetero HG-R 2	Fgf10	28.80756	28.81742949	13.76245602	7.1959E-05	1.73494					
57M Hetero HG-R 2	Fgf10	28.8273									
53M WT HG-L	Fgf7	29.65711	29.78625903	13.65923057	7.7297E-05	1.412844	WT L	5.45E-05	3.18E-05	1	0.58385
53M WT HG-L	Fgf7	29.91541									
54M WT HG-L	Fgf7	31.12406	31.075683	14.95359422	3.1515E-05	0.587156					
54M WT HG-L	Fgf7	31.02731									
53M WT HG-R	Fgf7	29.09981	29.05269437	14.22124465	5.2357E-05	0.954128	WT R	6.35E-05	1.63E-05	1.165138	0.298413
53M WT HG-R	Fgf7	29.00558									
54M WT HG-R	Fgf7	28.86243	28.78784555	13.69792226	7.5251E-05	1.376147					
54M WT HG-R	Fgf7	28.71326									
55M Hetero HG-L	Fgf7	31.11473	31.14090209	13.78056044	7.1062E-05	1.302752	Hetero L	5.97E-05	1.63E-05	1.094802	0.298883
55M Hetero HG-L	Fgf7	31.16707									
56M Hetero HG-L	Fgf7	30.11726	30.04017912	13.87626453	6.6501E-05	1.229358					
56M Hetero HG-L	Fgf7	29.9631									
57M Hetero HG-L	Fgf7	31.91396	32.01145469	14.58045238	4.0818E-05	0.752294					
57M Hetero HG-L	Fgf7	32.10895									
55M Hetero HG-R	Fgf7	34.53483	34.26554452	4.723860345	0.0378422						
55M Hetero HG-R	Fgf7	33.99626									
56M Hetero HG-R	Fgf7	28.67497	28.56495451	13.64132607	7.8262E-05	1.431193	Hetero R	0.000106	3.96E-05	1.944954	0.726569
56M Hetero HG-R	Fgf7	28.45494									
57M Hetero HG-R 2	Fgf7	28.01284	27.92404272	12.86906926	0.00013367	2.458716					
57M Hetero HG-R 2	Fgf7	27.83524									

Table S9.	List of animals/embryos shown in Figures.				
**Age/stage**	**Genotype**	**Sex**	**Animal #**	**Figure #**	**Experiments**
74w	WT	♂	3M	Figure 1A, B, E~G	HE, Masson
74w	*Fgf10+/-*	♂	11M	Figure 1C, C', D, H~J	HE, Masson
74w	*Fgf10+/-*	♂	5M	Figure S1A~C	HE, Masson
P18	*Fgf10+/-*	♂	40F_7	Figure 1N~Q	Masson, TUNEL
P18	WT	♂	26F_1	Figure S2B	Photography of the face
P18	*Fgf10+/-*	♀	26F_7	Figure S2C	Photography of the face
P14	WT		1	Figure 1R, S	Pancytokeratin
P14	*Fgf10+/-*		4	Figure 1T, U	Pancytokeratin
P14	WT		2	Figure S3A, B	Pancytokeratin
P14	*Fgf10+/-*		6	Figure S3C, D	Pancytokeratin
P14	*Fgf10+/-*		3 (2_3)	Figure S1D~F	HE, Masson
P10	*Fgf10+/-*		3	Figure S1G~J'	HE, Masson
P6	*Fgf10+/-*		2	Figure 1V, W	HE
P6	*Fgf10+/-*		2	Figure 1Z~Z"; Figure S4A~D"; Figure S5A~D	TUNEL
P6	*Fgf10+/-*		3	Figure 1X, X', Y	HE
P6	*Fgf10+/-*		3	Figure 1AA, AA'; Figure S4E-H; Figure S5E, F	TUNEL
P0.5	WT		11	Figure 2I, J	HE
P0.5	WT		6	Figure S3E, F	HE
P0.5	*Fgf10+/-*		10	Figure 2K, L	HE
P0.5	*Fgf10+/-*		5	Figure S3G, H	HE
P0.5	WT		11	Figure 2M, N	Pancytokeratin
P0.5	WT		6	Figure S3I, J	Pancytokeratin
P0.5	*Fgf10+/-*		10	Figure 2O, P	Pancytokeratin
P0.5	*Fgf10+/-*		5	Figure S3K, L	Pancytokeratin
E19	*Fgf10+/-*		26F_3(HG+)	Figure 4E, F	ISH for *Fgf7*
E19	*Fgf10+/-*		26F_3(HG+)	Figure S11E, F	ISH for *Fgf7*
E19	*Fgf10+/-*		26F_3(HG+)	Figure S12I, I'	F-actin
E19	*Fgf10+/-*		26F_6(HG+)	Figure S11G, H	ISH for *Fgf7*
E19	*Fgf10+/-*		26F_7(no HG)	Figure 4G~L	ISH for F*gf7, Fgf10, Fgfr2b*
E19	*Fgf10+/-*		26F_7(no HG)	Figure S9	ISH for *Fgf10, Fgfr2b*
E19	*Fgf10+/-*		26F_7(no HG)	Figure S11I, J	ISH for *Fgf7*
E19	*Fgf10+/-*		26F_8(no HG)	Figure S11K, L	ISH for *Fgf7*
E19	*Fgf10+/-*		26F_8(no HG)	Figure S12A~H	ISH for *Fgf10, Fgfr2b*
E19	*Fgf10+/-*		26F_2(no HG)	Figure S12J, J'	F-actin
E15	WT		36F_1	Figure 2C, C'; Figure S6A, B, A'	HE
E15	WT		36F_3	Figure S6C, D	HE
E15	WT		61F_6	Figure S6 E, F, F'	HE
E15	*Fgf10+/-*		36F_4	Figure 2D, D'; Figure S6G, H, G'	HE
E15	*Fgf10+/-*		36F_6	Figure S6I, J, J'	HE
E15	*Fgf10+/-*		61F_3	Figure S6K, L	HE
E15	*Fgf10-/-*		61F_5	Figure S6M, N	HE
E15	*Fgf10-/-*		61F_7	Figure S6O, P	HE
